# An Epidemiological Survey of Sepsis in a Tertiary Academic Hospital from Southwestern Romania

**DOI:** 10.3390/medicina61040596

**Published:** 2025-03-26

**Authors:** Andra Grigorescu, Florentina Dumitrescu, Stefania Dorobantu, Adina Dragos, Andrei Pirvu, Mihaela Roskanovic, on behalf of the FUSE study, Ioana Streata, Mihai Ioana, Mihai G. Netea, Anca-Lelia Riza

**Affiliations:** 1 Human Genomics Laboratory, Functional Genomics Group, University of Medicine and Pharmacy of Craiova, 200349 Craiova, Romania; andra.grigorescu@radboudumc.nl (A.G.); adina.crgm@gmail.com (A.D.); andrei.crgm@gmail.com (A.P.); ioana.streata@umfcv.ro (I.S.); mihai.netea@radboudumc.nl (M.G.N.); anca.costache@umfcv.ro (A.-L.R.); 2 Department of Internal Medicine and Radboud Center for Infectious Diseases, Radboud University Medical Center, 6500 HB Nijmegen, The Netherlands; 3 Infectious Disease Department, University of Medicine and Pharmacy of Craiova, 200349 Craiova, Romania; florentina.dumitrescu@umfcv.ro (F.D.); drmikiroskanovic@gmail.com (M.R.); 4 Hospital for Infectious Diseases and Pneumology “Victor Babeş” Craiova, 200515 Craiova, Romania; 5 Regional Centre of Medical Genetics Dolj, County Clinical Emergency Hospital Craiova, 200642 Craiova, Romania; 6 Department of Immunology and Metabolism, Life & Medical Sciences Institute, University of Bonn, 531143 Bonn, Germany

**Keywords:** severe infection, sepsis, *Clostridium difficile*

## Abstract

*Background and Objectives*: Sepsis is one of the major causes of death in modern society. This study is part of the FUSE (Functional Genomics in Severe Sepsis) project under the Human Functional Genomics Romania initiative. Our aim was to assess the epidemiology of sepsis in a tertiary academic hospital in southwestern Romania. *Materials and methods:* The study enrolled 184 patients with severe infections between May 2017 and November 2019, following the Sepsis-2 guidelines (SIRS criteria). *Results:* The present cohort of community-acquired severe infections shows respiratory and urinary tract as main sites of severe infection. The demographic and clinical characteristics of this Romanian study group are in line with those of other severe infection European cohorts. However, the predominance of confirmed *Clostridium difficile* cases represents a strong deviation, raising significant concerns for the communities to which the patients belong. *Conclusions*: Sepsis, with its complex pathophysiology and clinical presentation, remains one of the most daunting global health issues. In our cohort, the high number of *Clostridium difficile* cases prompts high vigilance and immediate intervention.

## 1. Introduction

Sepsis is one of the major causes of death in modern society in both developed and developing countries. It is a complex clinical condition characterized by interconnecting pathophysiological processes. The dysregulation of the host response to infection leads to organ failure and death in a significant number of cases, often despite appropriate medical intervention. A recent large survey estimated a yearly occurrence of nearly 49 million cases of sepsis, accounting for 20% of the global mortality; the incidence has been shown to be on a steady rise [1,2,3].

Based on a 2016 Centre for Disease Control (CDC) campaign, almost 80% of sepsis cases emerge in community settings and develop from severe infection. However, the contribution of healthcare-associated infections should not be underestimated. Efforts are necessary to obtain better reporting rates and efficiently implement preventive measures. Therefore, it is crucial to determine the complex demographic, clinical, and setting-associated factors underlying severe infections, with the aim of limiting their potential to develop into sepsis [4,5,6].

In terms of causative microorganisms, the reported etiology of sepsis and the source of infection vary between countries, regions, and even local hospitals. The patterns of antibiotic resistance are also very different around the world in general, and in Europe in particular, with countries in Southern and Eastern Europe reporting higher resistance prevalence [7,8,9]. These differences require adjusted prophylactic and therapeutic approaches, for which appropriate information on the local epidemiology of sepsis and severe infections is crucial.

Comprehensive epidemiological data on severe sepsis of either community or healthcare origin [10] are lacking in Romania. Hospital-associated infections in Romania, including sepsis, are significantly underestimated, with official prevalence rates of only 0.20–0.25% [11]. A thorough literature search yielded limited, mostly single-center reports on descriptive epidemiology [11,12], etiology of healthcare-associated [13,14], or antimicrobial resistance [15] in Romania. We could not identify studies that addressed broader epidemiological data on sepsis or severe hospital infections in Romania, such as incidence rates, patient outcomes, or comparisons with other pathogens. There is even less data on community-acquired infections. Detailed insights into the effectiveness of current prevention and control strategies for sepsis and severe infections in Romanian hospitals are also lacking [16].

The aim of the present study was to assess the epidemiology of sepsis in a tertiary academic hospital in southwestern Romania. As the mortality rate of sepsis and severe infections remains high, such an epidemiological survey is very important for informing appropriate healthcare measures to prevent sepsis and severe infection and mitigate their effects when they occur.

## 2. Materials and Methods

### 2.1. Study Cohort

The study cohort included 184 patients admitted to the clinical ward of the Hospital for Infectious Diseases and Pneumology “Victor Babes” Craiova, Romania, between May 2017 and November 2019. Patients were enrolled based on the following criteria: aged 18 years and above, with a sepsis diagnosis according to the Sepsis-2 guidelines (SIRS criteria). Patients with a history of inherited or acquired immunodeficiency were excluded from the study.

The diagnosis was stratified according to the Sepsis-2 criteria: sepsis, severe sepsis, or septic shock. Sepsis diagnosis was established if at least two of the following criteria were met: (1). body temperature of >38 °C or <36 °C; (2). heart rate of >90 bpm; (3). respiratory rate of > 20/min or PCO_2_ < 32 mmHg; (4). Leukocytosis of >12.000/mm^3^ or leukopenia (<4.000/mm^3^) or >10% blasts. Severe sepsis was classified based on the presence of at least one condition, underlining organ failure, such as altered pulmonary (diffuse bilateral consolidation) or renal function (oliguria of <0.50 mL/kg/h) or acidosis, indicative of organ hypoperfusion, manifested in the forms of a pH < 7.30 or a base deficit > 5 mEq/L or lactate measurements twice more than the normal value. Septic shock was declared when the patient’s condition included a systolic blood pressure of less than 90 mmHg, mean arterial pressure < 60 mmHg, or the need for fluid replacement therapy or vasopressors [1].

### 2.2. Data Collection

Information related to lifestyle factors and past medical history was collected in the Infectious Diseases Clinic by attending physicians and recorded using a structured questionnaire. Clinical evaluation was likewise performed by the attending clinicians.

### 2.3. Sample Collection and Paraclinical Investigations

Hematologic and biochemistry tests were performed in the clinical laboratory of the hospital using samples collected during the hospital stay. Furthermore, biological samples, including blood, urine, feces, bronchoalveolar lavage (BAL) samples, nasal and pharyngeal exudates, cerebrospinal fluid, and peritoneal fluid, which were used for performing bacterial cultures and the corresponding antibiogram, were selected based on a suggestive clinical context. The nature and timepoint of culture collection respected the clinical rationale and internal procedures of the Infectious Diseases ward to which the patients were admitted.

Sample processing and bioarchiving were performed at the Human Genomics Laboratory of the University of Medicine and Pharmacy of Craiova. Serum ferritin measurements were performed at the Laboratory of Experimental Internal Medicine, Radboud University Medical Center. Sera were separated no further apart than 4 h from collection and long-term stored at −80 °C.

All blood samples were collected prior to the administration of any antibiotic therapy during the hospital stay.

### 2.4. Statistical Analysis

Statistical analysis was performed using R software version 4.3.2, packages dplyr, ggplot2, tidyr, tidyverse, rstatix, grid, gridExtra, magrittr, stringr, ggpubr, cowplot, plotly, FSA, stats, vcd, UpSetR, tibble and Cairo, both for data processing and generating the graph plots. The normal distribution of continuous data was evaluated using the Shapiro−Wilk test and Q-Q plots.

For normally distributed data, a two-way ANOVA test was employed, followed by Tukey’s HSD (honestly significant difference), with the aim of identifying the specific subgroups between which the difference is valid. Conversely, the non-parametric test choice was the Kruskal−Wallis test, followed by the Dunn-Bonferroni test as a post-hoc analysis. Categorical data were interpreted using Fisher’s exact test.

## 3. Results

### 3.1. Cohort Description

A total of 184 patients were included in the study. Sex distribution was approximately equal (53.26% females), and the median age was 64.50 years. Most of the patients lived in an urban environment (57.60%), with the rest living in rural villages in the Dolj county. The median BMI was 24.86, and 30.43% of the patients were smokers (Table 1).

According to the Sepsis-2 criteria, 129 subjects (70.10%) had sepsis, 51 had severe sepsis, and four had septic shock.

Depending on the source of infection, most patients suffered from *Clostridium difficile* enterocolitis (21.73%), followed by infections of the lower respiratory tract, mainly community-acquired pneumonia (pneumosepsis, 20.65%), and severe infections of the urinary tract represented by pyelonephritis (urosepsis, 19.02%). Other notable infection subgroups included ear, nose, and throat (ENT) infection sources and other digestive infections different from *Clostridium difficile*. Seventeen patients had clinical features that overlapped with those of the main severe infection subgroups.

We subsequently investigated the demographic and lifestyle parameters of the sepsis phenotypes described above. Among the findings, unexpectedly, we observed a higher median age in the *Clostridium difficile* patient subgroup and a higher percentage of women among patients with urosepsis (Table 1). Smoking was more frequent in patients with severe respiratory infections. A more detailed characterization of the demographic and lifestyle parameters of the study cohort and the main infection subgroups is presented in Table 1 and in the Supplementary Data.

### 3.2. Medical History

**Figure 1:**
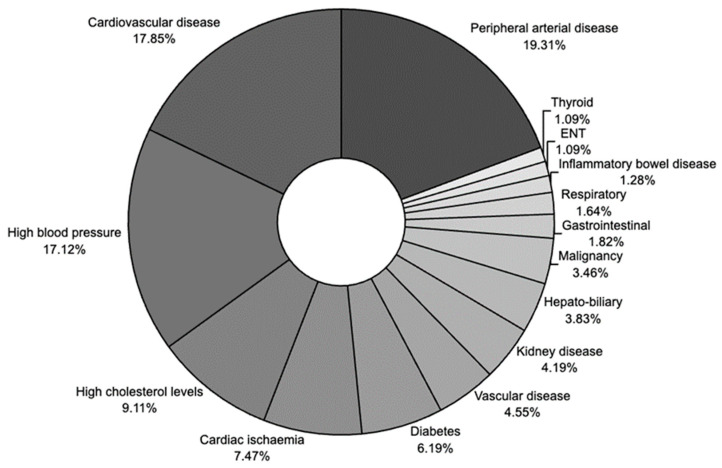
Distribution of comorbidities in the FUSE severe infection cohort.

The prevalent comorbidities identified in our sepsis cohort were peripheral arterial disease (19.30%), cardiovascular disease (17.90%), and high blood pressure (17.10%), with no differences in these comorbidities between the three main severe infection subgroups (Table 2, Figure 1). In contrast, chronic kidney disease was more prevalent in the urinary severe infection subgroup. A breakdown of the comorbidity distribution within the severe infection cohort is presented in the Supplementary Material. Notably, in our cohort, 41.60% of patients had a documented prior admission within a three-month timeframe, and a similar percentage reported recent antibiotic use.

Table 3 presents the paraclinical characteristics of the cohort.

As expected, a high percentage of *Clostridium difficile* patients (80.00%) had recently used antibiotics.

### 3.3. Bacterial Etiology

**Figure 2:**
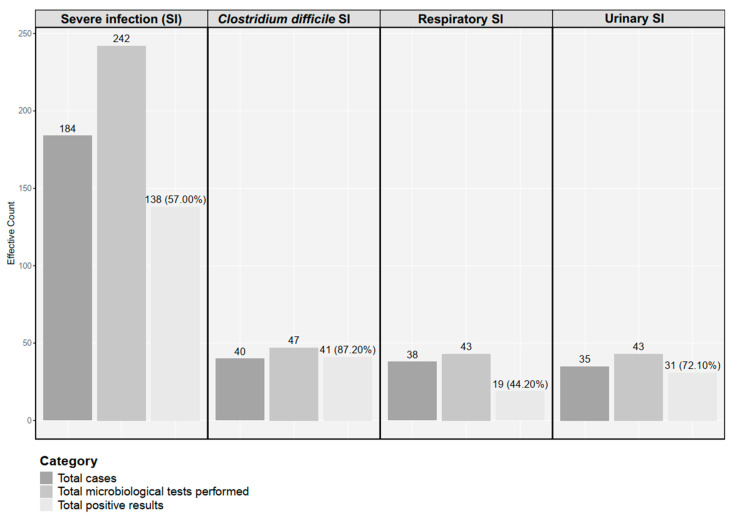
Microbiological testing in the whole severe infection cohort, as well as stratified by the main infection subgroups. SI—severe infection.

A total of 242 cultures were performed for 184 patients, with 138 positive results (57.00%). Figure 2 illustrates the number of microbiological tests performed for the main sepsis subgroups and the corresponding number of positive results. At least one microbiological test was performed for each patient included in the cohort.

**Figure 3:**
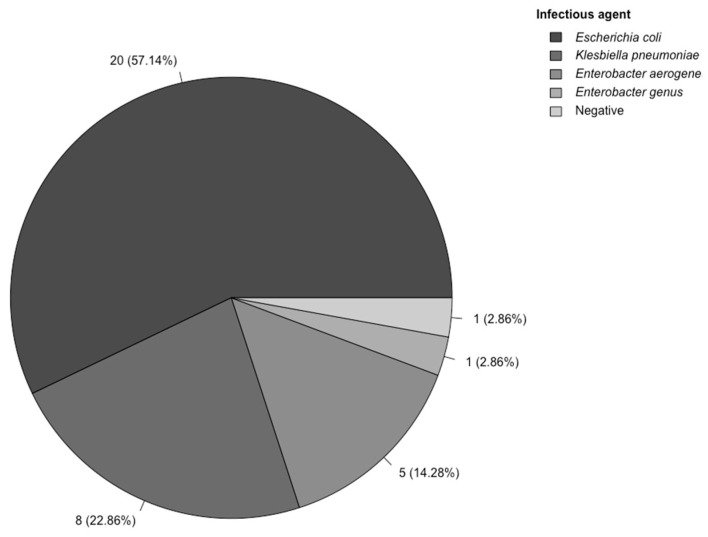
Infectious agents identified in urine cultures - severe urinary tract infection subgroup.

The most frequent infectious agents identified in our study cohort were *Clostridium difficile* (35.80%), *Escherichia coli* (21.50%), *Klebsiella pneumoniae* (11.50%), and *Staphylococcus aureus* (5.40%). Figure 3 focuses on the main infectious agents identified in urine cultures in the severe urinary infection subgroup.

The status of each microbiological test performed, including sputum, urine, blood, purulent material, and *other* culture types (feces, nasal and pharyngeal exudates, cerebrospinal fluid, and peritoneal fluid), is presented in the Supplementary Material.

The highest rate of positive results was identified for the microbiological tests performed for *Clostridium difficile*, followed by cultures performed from urine (62.85%). Notably, a low rate of positive results was observed for sputum samples (12.50%).

We subsequently assessed the status of positive blood cultures in relation to the main sepsis phenotypes and investigated the overlap between the causative infectious agents. Blood cultures were collected for 18.47% of cases, with a culture-positive rate of 38.23%. For the *Clostridium difficile* subgroup, only two blood cultures yielded positive results, both with *Staphylococcus aureus*. In the respiratory severe infection subgroup, all six blood cultures performed had negative results. The urinary severe infection subgroup presented four positive blood cultures, with *Escherichia coli* being the etiological cause in all.

Our investigation further evaluated the antibiogram data, which indicated that the highest overall resistance was registered for the combination of ampicillin−sulbactam (33.30%), with a susceptibility of 18.20%. Generally, a higher level of resistance was reported for extended-spectrum penicillins.

Narrowing down the antibiogram data for the main infectious agents identified for our study cohort, we sought to characterize the antibiotic susceptibility and resistance of *Escherichia coli*, *Klebsiella pneumoniae*, and *Staphylococcus aureus*, irrespective of the culture type or infection source. Notably, the combination of trimethoprim−sulfamethoxazole was correlated with high resistance levels for *Klebsiella pneumoniae* (52.17%); however, it was extracted from seven of ten test results. A certain level of resistance (17.41–21.36%) to extended-spectrum penicillins was reported for both *Escherichia coli* and *Klebsiella pneumoniae*. Quinolones and fluoroquinolones exhibited resistance levels of 4.54–9.23%. Unfortunately, we had limited data for an accurate *Staphylococcus aureus* antibiogram analysis, as only six antibiograms were available for this particular pathogen.

### 3.4. Antibiotic Therapy During Hospitalization

The most frequent antibiotic regimen employed for the patients in our cohort included either beta-lactam cephalosporins (including oxacephem), glycopeptides, or beta-lactam penicillin. For the Clostridium difficile enterocolitis subgroup, glycopeptides, in the form of vancomycin, were the leading therapeutic agents. Beta-lactam cephalosporins were the preferred choice for both the respiratory and urinary subgroups, followed by carbapenems and penicillin, respectively. The extended picture of the antibiotic course is presented in the Supplementary Material.

### 3.5. Outcome of the Severe Infection

**Figure 4:**
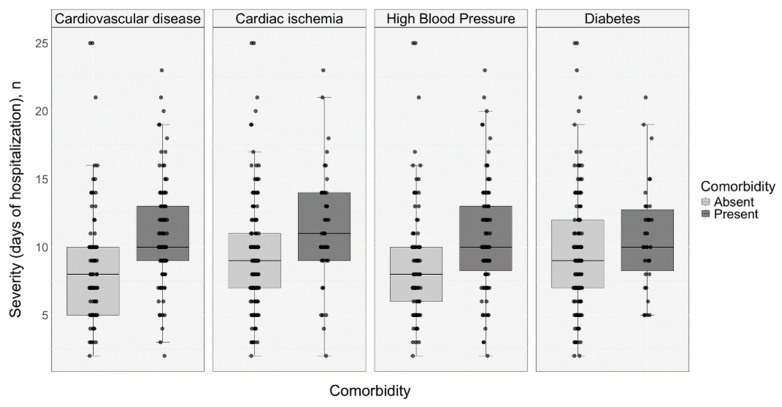
Comorbidity distribution within the severe infection cohort, stratified by days of hospitalization (*p* < 0.05).

The median length of hospital stay was 9.00 days for the entire cohort. Cardiovascular disease, cardiac ischemia, high blood pressure, and diabetes were associated with a moderately prolonged hospital stay and a statistically significant correlation (*p* < 0.05). (Figure 4).

Mortality in our study was low. Severe infection was the cause of death for three patients included in the study cohort: cardiac and respiratory failure was the cause of death in a female, aged 91 years, with severe *Staphylococcus aureus* infection; severe *Staphylococcus aureus* and *Serratia marcescens* infection in a 75-year-old male; and kidney failure in an 86-year-old male suffering from a severe form of *Clostridium difficile* infection.

## 4. Discussion

In the present study, we assessed the demographic, etiological, and severity parameters of patients with sepsis in a tertiary university hospital in southwestern Romania. Approximately 200 patients fulfilling the Sepsis-2 criteria were included in the study between May 2017 and November 2019. Most patients developed sepsis as a consequence of respiratory and urinary tract infections contracted in a community setting, while a significant minority presented with *Clostridium difficile* infection and a recent history of hospitalization. Gram-negative bacteria were the main probable etiological cause of the severe infection cohort, and antibiotic treatment reflected a significant prevalence of antibiotic resistance; however, in the absence of corresponding blood culture results, the definitive sepsis etiology is difficult to pinpoint.

Despite the high incidence and severity of the disease, little is known about the etiology, treatment, and outcomes of sepsis in Romanian hospitals. While the sepsis cohort presented in the current study was recruited from a single university hospital in Romania, it does provide important information about the particularities of patients with sepsis in the country. Thus, on the one hand, the main sources of the infection (respiratory tract, urinary tract, digestive tract) do not differ significantly from other countries. In contrast, a clear particularity with respect to the etiological agents identified in this cohort is the apparent predominance of Gram-negative bacteria. A similar predominance of Gram-negative microorganisms has been reported in other Southern and Eastern European countries [17,18]. In contrast, in most Northern and Western European countries, Gram-positive bacteria, especially *Staphylococcus aureus*, are the main etiological cause of sepsis [19,20,21]. The underlying cause of this difference is not completely clear, although important differences in antibiotic policy are likely to drive these differences, at least in part.

An important aspect that needs particular attention is the very high prevalence of *Clostridium difficile* infection in our cohort. *Clostridium difficile* infections often follow antibiotic treatment in hosts with weakened host defenses [22,23,24], and they can become a particular challenge for hospitalized patients if appropriate measures are not taken to contain the spread of these infections [25,26,27]. *Clostridium difficile* infections are the most common cause of hospital-acquired infectious diarrhea in developed countries, and their incidence has increased in recent years due to the emergence of a hypervirulent strain of the microorganism—the North American pulsed-field type 1 NAP1/PCR ribotype 027 [28]. As this strain has been identified in many countries in both North America and Europe, it would be crucial to investigate whether the same strain is responsible for the high prevalence of *Clostridium difficile* infection in Romania. Subsequently, studies in additional Romanian hospitals need to be conducted to assess the prevalence of this infection and the novel hypervirulent strain, and aggressive preventive measures should be implemented to restrict its spread.

The demographic characteristics of Romanian patients with sepsis are relatively similar to those of other cohorts, including the presence of comorbidities. A median age of 64.5 years reflects the increased susceptibility to infections of individuals of older age. More surprising is the higher percentage of women than men in our cohort (57.00%), which is different from the known higher susceptibility to infections in men [29,30]. At least part of the cause for this difference may be the relatively high prevalence of urosepsis, with a predominance in women, and the fact that Sepsis-2 rather than Sepsis-3 criteria were used for diagnosis in our cohort, resulting in a less severely ill population. This last aspect is most likely responsible for the low mortality rate in our cohort.

An important aspect to be considered is the treatment of patients with regimens that contain many late-generation antibiotics. This is due to the high prevalence of antibiotic resistance, which has been highlighted by other earlier surveys [8,15,31]. This is a particular point of concern that necessitates concerted action, from a rigorous application of antibiotic guidelines to increased education of both medical personnel and the general population on the importance of strict antibiotic policy and the need for judicious use of such treatments.

Our study also has a number of limitations. One limitation is the absence of the SOFA score calculation and, therefore, the impossibility of classifying our patients based on the Sepsis-3 criteria. In light of previous publications, the Sepsis-3 criteria aim to simplify the sepsis classification process, with a higher specificity but lower sensitivity [32,33]. A second limitation is the single-center nature of our study; thus, our findings need to be replicated in large-scale research initiatives.

In conclusion, we present the demographic and clinical characteristics of a cohort of patients with sepsis from a tertiary university center in Romania. Important characteristics of Romanian patients with sepsis include the predominance of Gram-negative bacteria as a probable etiological cause of sepsis, a high prevalence of *Clostridium difficile* infection, and a high prevalence of antibiotic resistance. However, the mortality was low due to the use of Sepsis-2 diagnostic criteria, and future surveys need to extend both Sepsis-3 criteria and multiple hospitals. Such future studies will be crucial for applying the appropriate prophylactic and therapeutic measures to improve the outcomes of patients with sepsis.

## 5. Conclusions

Sepsis, with its complex pathophysiology and clinical presentation, remains one of the most daunting global health issues that need to be collectively addressed, for which extended data are needed. The present cohort of community-acquired severe infections, part of the FUSE study, reaffirms the characteristics of previous severe infection cohorts for the Romanian population in relation to demographic parameters and clinical aspects, in line with other European studies. However, the predominance of confirmed *Clostridium difficile* cases represents a strong deviation from known results, signaling severe issues rooted in the healthcare setting and raising significant concerns for the communities to which the patients belong. This prompts high vigilance and immediate intervention from local public health authorities and all medical personnel.

## Figures and Tables

**Table 1:** FUSE severe infection cohort—demographic and lifestyle data.

Characteristics		Severe Infection Cohort (*n* = 184)	*Clostridium difficile* Severe Infection *n* = 40		Respiratory Severe Infection *n* = 38		Urinary Severe Infection *n* = 35		*p*-Value ^2^	Post-hoc Tests ^3^		
				*p*-Value ^1^		*p*-Value ^1^		*p*-Value ^1^		C vs. R	C vs. U	R vs. U
**Age median (IQR)**		64.5 (39.00–75.00)	67.5 (56.50–72.75)	-	64 (46.50–75.00)	-	65 (30.00–74.00)	-	0.65	NA	NA	NA
**Age groups,** ***n* (%)**	**18–30**	32 (17.39%)	5 (12.50%)	-	5 (13.15%)	-	10 (28.57%)	-	0.27	NA	NA	NA
	**31–45**	25 (13.58%)	4 (10.00%)	-	5 (13.15%)	-	3 (8.57%)	-	0.12	NA	NA	NA
	**46–60**	22 (11.95%)	2 (5.00%)	-	5 (13.15%)	-	4 (11.42%)	-	0.66	NA	NA	NA
	**>60**	105 (57.06%)	29 (72.50%)	-	23 (60.52%)	-	18 (51.42%)	-	0.31	NA	NA	NA
**Sex, female, *n* (%)**		98 (53.26%)	18 (45.00%)	0.28	21 (55.26%)	0.86	21 (60.00%)	0.45	-	-	-	-
**Background, urban (*n*, %)**		106 (57.60%)	23 (57.50%)	1.00	20 (52.63%)	0.58	18 (51.42%)	0.45	-	-	-	-
**BMI median (IQR)**		24.86 (21.93–27.43)	22.31 (19.28–25.86)	-	23.90 (22.40–28.16)	-	25.09 (22.22–26.50)	-	0.04	0.07	0.09	1.00
**Smoking (either present or past), *n* (%)**		56 (30.43%)	8 (20.00%)	0.12	17 (44.73%)	0.05	6 (17.14%)	0.07	-	-	-	-

^1^ *p*-value (Fisher’s exact test for categorical data, *t*-test as parametric test, Kruskal−Wallis as non-parametric test)—reflects the comparison of the subgroup of severe infection vs. the rest of the cohort. ^2^ Kruskal−Wallis/two-way ANOVA. ^3^ *p* adjusted, Dunn-Bonferroni/Tukey’s HSD. *n*—number of cases. IQR—interquartile range. C—Clostridium difficile severe infection group. R—respiratory severe infection group. U—urinary severe infection group. NA—not applicable. The data distribution (not shown) was calculated for continuous variables using the Shapiro−Wilk test and showed no deviation from the normal distribution.

**Table 2:** FUSE severe infection cohort—clinical history

Characteristics		Severe Infection Cohort *n* = 184	*Clostridium difficile* Severe Infection *n* = 40		Respiratory Severe Infection *n* = 38		Urinary Severe Infection *n* = 35		*p*-Value ^2^
				*p*-Value ^1^		*p*-Value ^1^		*p*-Value ^1^	
**Past antibiotic use, *n* (%)**		75 (40.76)	32 (80.00%)	1.48 × 10^−5^	12 (31.57%)	0.58	11 (31.42%)	0.19	-
**DM treatment, *n* (%)**	**Type II, insulin, *n* (%)**	6 (3.26%)	2 (5.00%)	NA	0	NA	1 (2.85%)	NA	-
	**Number of years any diabetes treatment—median (IQR)**	5 (3.00–11.00)	10 (4.00–11.00)	-	7.5 (6.25–8.75)	-	5 (0.51–19.50)	-	0.95
**Glucocorticoid use**		18 (9.78%)	3 (7.50%)	0.77	4 (10.52%)	0.77	2 (5.71%)	0.53	-
**Medical history, *n* (%)**	**Cardiovascular disease**	98 (53.26%)	25 (62.50%)	0.21	25 (65.78%)	0.10	16 (45.71%)	0.35	-
	**Vascular disease**	25 (13.58%)	2 (5.00%)	0.11	7 (18.42%)	0.42	6 (17.14%)	0.58	-
	**High blood pressure**	94 (51.09%)	24 (60.00%)	0.22	24 (63.15%)	0.10	16 (45.71%)	0.57	-
	**Cardiac ischemia**	41 (22.28%)	10 (25.00%)	0.67	11 (28.94%)	0.28	7 (20.00%)	1.00	-
	**High cholesterol levels**	50 (27.17%)	12 (30.00%)	1.00	15 (39.47%)	0.06	10 (28.57%)	1.00	-
	**Peripheral arterial disease**	106 (57.60%)	25 (62.50%)	0.59	26 (68.42%)	0.14	21 (60.00%)	0.85	-
	**Kidney disease**	23 (12.50%)	4 (10.00%)	0.60	3 (7.89%)	0.42	10 (28.57%)	<0.01	-
	**Diabetes**	34 (18.47%)	9 (22.50%)	0.49	3 (7.89%)	0.06	7 (20.00%)	0.81	-
	**Inflammatory bowel disorder**	7 (3.80%)	2 (5.00%)	0.66	0	0.35	2 (5.71%)	0.64	-
	**Malignancy**	19 (10.32%)	9 (22.50%)	0.07	2 (5.26%)	0.37	3 (8.57%)	1.00	-
	**Gastrointestinal**	10 (5.43%)	2 (5.00%)	NA	3 (7.89%)	NA	1 (2.85%)	NA	-
	**Hepato-biliary**	21 (11.41%)	6 (15.00%)	NA	3 (7.89%)	NA	4 (11.42%)	NA	-
	**Respiratory**	9 (4.89%)	4 (10.00%)	NA	3 (7.89%)	NA	0	NA	-
	**ENT**	6 (3.26%)	1 (2.50%)	NA	2 (5.26%)	NA	0	NA	-
	**Thyroid**	6 (3.26%)	3 (7.50%)	NA	0	NA	1 (2.85%)	NA	-
**Previous hospitalization, *n* (%)**	**Hospital admission 3 months prior to enrollment**	76 (41.30%)	30 (75.00%)	1.36 × 10^−6^	12 (31.57%)	0.20	9 (25.71%)	0.06	

^1^ *p*-value (Fisher’s exact test for categorical data, *t*-test as parametric test, Kruskal Wallis as non-parametric test)—reflects the comparison: subgroup of severe infection vs. rest of the cohort. ^2^ Kruskal Wallis/two-way ANOVA. *n*—number of cases. IQR—interquartile range. NA—not applicable. The data distribution (not shown) was calculated for continuous variables using the Shapiro−Wilk test and showed no deviation from the normal distribution.

**Table 3:** FUSE severe infection cohort—paraclinical characteristics.

Median (IQR)		Severe Infection Cohort n = 184	*Clostridium difficile* Severe Infection *n* = 40		Respiratory Severe Infection *n* = 38		Urinary Severe Infection *n* = 35		*p*-Value ^2^	Post-Hoc Tests ^3^		
				*p*-Value ^1^		*p*-Value ^1^		*p*-Value ^1^		C vs. R	C vs. U	R vs. U
**Leukocyte count**	**Total (1000/mm^3^)**	15.45 (13.05–19.47)	17.40 (13.72–20.07)	-	14.20 (7.72–17)	-	16.20 (13.50–20.45)	-	0.07	NA	NA	NA
	**Neutrophils, segmented (%)** missing values: 1	74.00 (67.00–79.00)	70.50 (65.50–77.5)	-	73.00 (65.25–8.75)	-	76.00 (67.00–84.00)	-	0.28	NA	NA	NA
	**Monocytes (%)** missing values: 1	5.00 (4.00–7.00)	6.00 (4.00–8.00)	-	6.00 (4.25–7.75)	-	5.00 (3.00–7.00)	-	0.54	NA	NA	NA
	**Lymphocytes (%)** missing values: 1	10.00 (6.00–15.00)	11.00 (6.75–18.25)	-	12.00 (7.00–19.50)	-	8.00 (5.00–13.00)	-	0.06	NA	NA	NA
**Inflammation**	**ESR 2 h** missing values: 9	75.00 (50.00–105.00)	70.00 (55.00–90.00)	-	80.50 (42.75–110)	-	83.50 (52.75–111.50)	-	0.44	NA	NA	NA
	**Ferritin** missing values: 24	198.96 (87.63–324.10)	224.81 (144.02–362.69)	-	175.07 (74.81–300.00)	-	166.43 (71.82–263.92)	-	0.27	NA	NA	NA

^1^ *p*-value (Fisher’s exact test for categorical data, *t*-test as parametric test, Kruskal Wallis as non-parametric test)—reflects the comparison: subgroup of severe infection vs. rest of the cohort. ^2^ Kruskal−Wallis/two-way ANOVA. ^3^ *p* adjusted, Dunn-Bonferroni/Tukey’s HSD could not be computed as *p*-value was not significant. *n*—number of cases. IQR—interquartile range. C—Clostridium difficile severe infection group. R—respiratory severe infection group. U—urinary severe infection group. NA—not applicable. The data distribution (not shown) was calculated for continuous variables using the Shapiro−Wilk test and showed no deviation from the normal distribution.

## Data Availability

The datasets generated and/or analyzed during the current study are available from the corresponding author upon reasonable request.

## Supplementary Materials

The following supporting information can be downloaded at: https://www.mdpi.com/article/10.3390/medicina61040596/s1, Figure S1. Age and sex distribution within the severe infection cohort, stratified by the main infection subgroups. Figure S2. BMI distribution within the severe infection cohort, stratified by the main infection sites. Figure S3. Urban/rural distribution within the severe infection cohort, stratified by the main infection sites. Figure S4. Smoking status distribution within the severe infection cohort, stratified by the main infection sites. Figure S5. Age and sex distribution within the severe infection cohort stratified by sepsis severity. Figure S6. BMI distribution within the severe infection cohort, stratified by severity. Figure S7. Urban/rural distribution within the severe infection cohort stratified by sepsis severity. Figure S8. Smoking status distribution within the severe infection cohort, stratified by sepsis severity. Figure S9. Number of microbiological tests performed per patient for the severe infection cohort and main infection subgroups. Figure S10. Main microbiological tests performed in the severe infection cohort. Figure S11. Main antibiotic classes used during hospitalization for the severe infection cohort). Figure S12. Main antibiotic classes used during hospitalization in the severe urinary infection subgroup). Figure S13. Main antibiotic classes used during hospitalization for the severe urinary infection subgroup).
