# Supplementary material for: An Epidemiological Survey of Sepsis in a Tertiary Academic Hospital from Southwestern Romania
Source: Medicina (Kaunas). 2025 Mar 26;61(4):596. doi: 10.3390/medicina61040596 (PMC12028556; doi:10.3390/medicina61040596)
Supplement: Supplementary file 1 [file medicina-61-00596-s001.zip › medicina-3513147-S1.pdf]

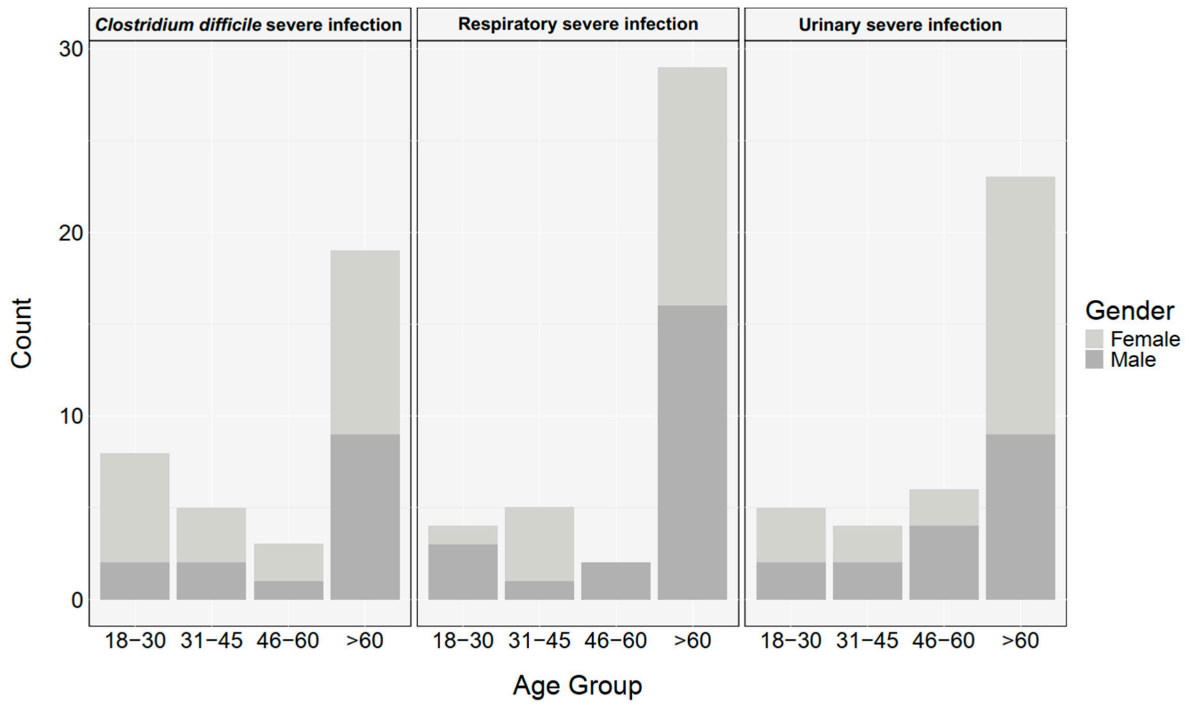

Figure S1. Age and gender distribution within the severe infection cohort, stratified by the main infection subgroups
