# Supplementary figures and images for: An Epidemiological Survey of Sepsis in a Tertiary Academic Hospital from Southwestern Romania
Source: Medicina (Kaunas). 2025 Mar 26;61(4):596. doi: 10.3390/medicina61040596 (PMC12028556; doi:10.3390/medicina61040596)

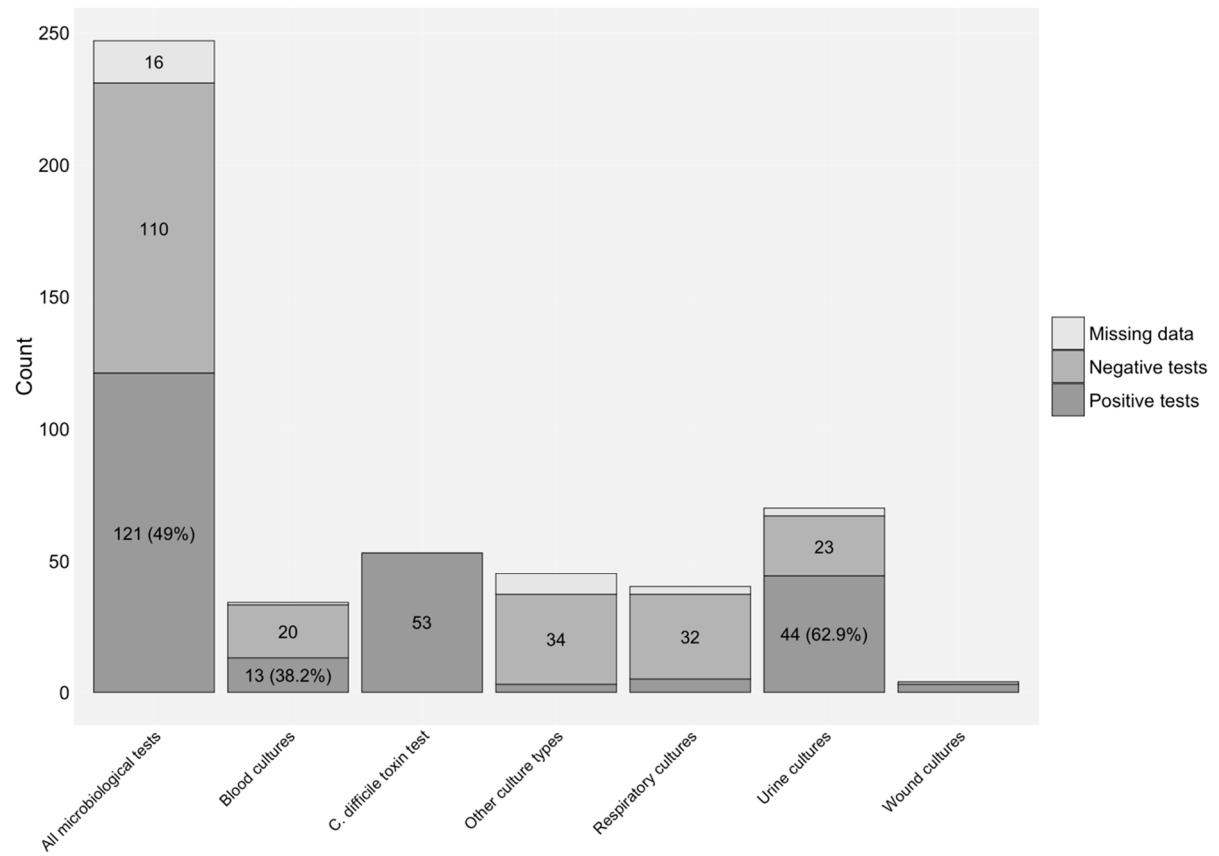

**Figure S10.** Main microbiological tests performed for the severe infection cohort

Supplement: Supplementary file 1 [file medicina-61-00596-s001.zip › medicina-3513147-S10.pdf]

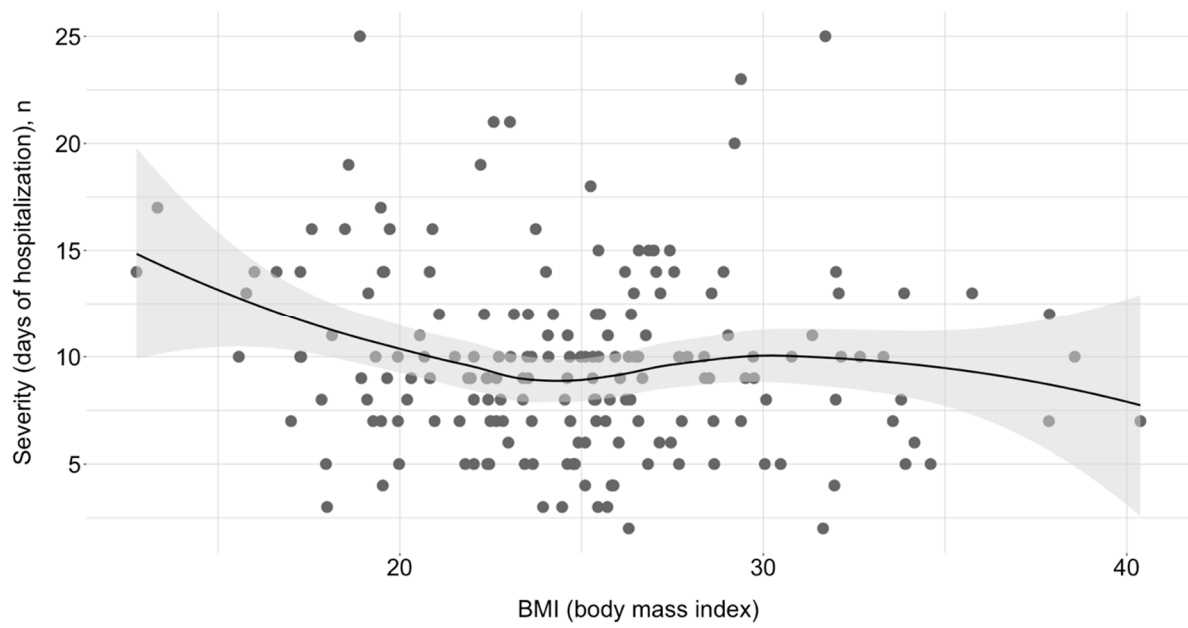

**Figure S6.** BMI distribution within the severe infection cohort, stratified by severity

Supplement: Supplementary file 1 [file medicina-61-00596-s001.zip › medicina-3513147-S6.pdf]

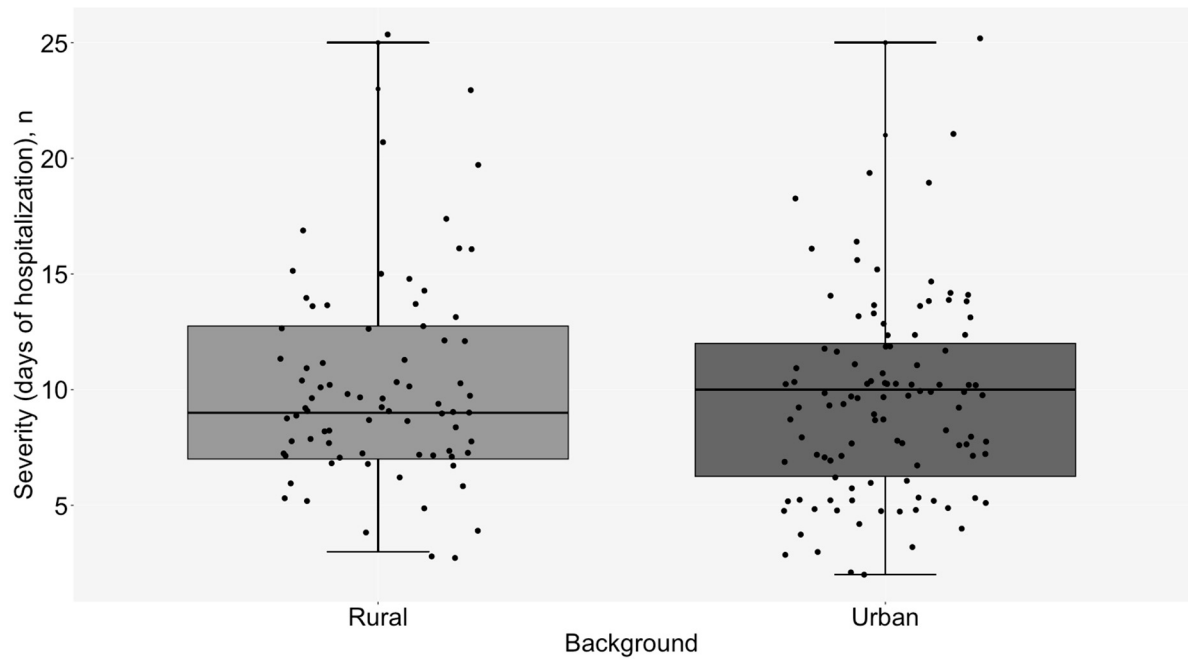

**Figure S7.** Urban/ rural distribution within the severe infection cohort, stratified by severity

Supplement: Supplementary file 1 [file medicina-61-00596-s001.zip › medicina-3513147-S7.pdf]
